# Hydration Shells of DNA from the Point of View of Terahertz Time-Domain Spectroscopy

**DOI:** 10.3390/ijms222011089

**Published:** 2021-10-14

**Authors:** Nadezda A. Penkova, Mars G. Sharapov, Nikita V. Penkov

**Affiliations:** 1 Institute of Theoretical and Experimental Biophysics, Russian Academy of Sciences, 142290 Pushchino, Russia; kokanchik@rambler.ru; 2 Institute of Cell Biophysics RAS, Federal Research Center “Pushchino Scientific Center for Biological Research of the Russian Academy of Sciences”, 142290 Pushchino, Russia; sharapov.mg@yandex.ru

**Keywords:** THz-TDS, hydration shells, DNA hydration, water structure, dielectric properties

## Abstract

Hydration plays a fundamental role in DNA structure and functioning. However, the hydration shell has been studied only up to the scale of 10–20 water molecules per nucleotide. In the current work, hydration shells of DNA were studied in a solution by terahertz time-domain spectroscopy. The THz spectra of three DNA solutions (in water, 40 mm MgCl_2_ and 150 mM KCl) were transformed using an effective medium model to obtain dielectric permittivities of the water phase of solutions. Then, the parameters of two relaxation bands related to bound and free water molecules, as well as to intermolecular oscillations, were calculated. The hydration shells of DNA differ from undisturbed water by the presence of strongly bound water molecules, a higher number of free molecules and an increased number of hydrogen bonds. The presence of 40 mM MgCl_2_ in the solution almost does not alter the hydration shell parameters. At the same time, 150 mM KCl significantly attenuates all the found effects of hydration. Different effects of salts on hydration cannot be explained by the difference in ionic strength of solutions, they should be attributed to the specific action of Mg^2+^ and K^+^ ions. The obtained results significantly expand the existing knowledge about DNA hydration and demonstrate a high potential for using the THz time-domain spectroscopy method.

## 1. Introduction

Water is well known as a substance of the highest importance for the stability and functioning of biological macromolecules. Deoxyribonucleic acid (DNA) is not an exception. Decrease of hydration degree below certain values causes dramatic structural rearrangements in the DNA molecule [1,2,3,4]. W. Saenger put forward the so-called “hydration economy concept” to explain this phenomenon [5]. According to the concept, DNA is arranged under water deficiency conditions in order to maintain the maximal hydration degree of phosphate groups via sharing the available water molecules.

Different methods have been used to study DNA hydration, such as gravimetry [6], IR spectroscopy [7,8], NMR [9], inelastic neutron scattering [10], densitometric and compressibility measurement methods [11], computer simulation [12,13], etc. The overall conclusion of the studies in this area can be expressed as follows: a hydration shell containing 10–20 water molecules per nucleotide is necessary to maintain a native DNA structure. It should be noted that most of the mentioned studies were carried out on weakly hydrated DNA films. A significantly lower number of works were dedicated to studies of DNA in solution, although such a system is more relevant to native DNA.

Dielectric spectroscopy is one of the most fruitful methods for studying the hydration properties of DNA in solutions [14,15,16,17,18]. It allows determining complex dielectric permittivity (DP). Different modifications of this method cover a vast range of frequencies: 1–10^12^ Hz. Dielectric spectroscopy provides information on the collective dynamics of water molecules, ions and the DNA molecule itself. Several dispersion ranges were identified for the dielectric permittivity of DNA solutions. Specific frequencies of rotational relaxation of DNA molecules are observed over the range of 100–1000 Hz [15]. The 1–10 kHz range corresponds to the process of dielectric relaxation related to the polarization of ionic shells of DNA molecules [15,16]. The area of around 100 MHz was attributed to the relaxation of clusters of bound water molecules. Meanwhile, the stability of these clusters was shown to be impaired above the melting point [14,18]. Dispersion around 20 GHz is related to Debye relaxation of water [14,17,18], and it depends on the binding degree of DNA hydration shells.

Terahertz time-domain spectroscopy (THz-TDS) is a relatively new method capable of DP determination [19,20]. It allows widening the spectral range of dielectric spectroscopy capability to higher frequencies, up to 3–4 THz. The terahertz range is located between the gigahertz region, where collective dynamics of substance molecules are observed, and the infrared region, where intramolecular oscillations are detected. Frequencies and energies of the terahertz region correspond to the intermolecular structure and dynamics of water molecules [21], which is directly related to studying hydration shells.

Currently, there are only a few studies on the application of THz spectroscopy for studying the hydration shells of DNA in solutions. In one work [22], the THz spectra of water solutions of nucleotides were measured in the range from 0.15 to 1.5 THz. Purine nucleotides decelerated the relaxation dynamics of water molecules, while pyrimidine nucleotides caused their acceleration in concentrations up to 1 mM. In [23], an analysis of the dielectric functions in the range of 0.4–1.6 THz allowed us to conclude that the relaxation dynamics of water molecules in DNA solutions are slowed down. Another paper [24] was dedicated to studying DNA solutions (0.5–20 mg/mL) in the range from 0.1 to 1.4 THz. Based on the concentration dependencies of the THz spectra, the thickness of DNA hydration shells was assumed to be around 16 Å. Meanwhile, it deserves mentioning that the suspension model of the effective medium used in the work was initially developed for the analysis of clay–sand slurry [25], and its analogy with DNA solution is absolutely unclear. In another work [26], the authors concluded that there were no differences between DNA solution and water in the parameters of relaxation processes calculated from spectra in the range of 0.3–2.1 THz. It might be related to the DNA concentration used in the work (<1 mg/mL), which is too low to detect any effects.

The main difficulties in the application of THz spectroscopy for studying water solutions of DNA and any other biological molecules are due to very strong absorption of water compared to DNA, blurring of spectral bands and non-characteristicity of the spectra. To obtain informative data on hydration shells, high-precision spectral measurement and specific approaches to data processing are required. In some of our works, the THz-TDS method has already been applied to study electrolyte solutions [27], protein hydration shells [28], liposomes [29], and ATP [30]. This work is the next step in this series of research articles, and it is focused on THz-TDS application to DNA solutions. The potential of the THz-TDS method in the 0.3–3.3 THz range was exploited to study DNA hydration shells and the influence of two basic cations of the intracellular medium, K^+^ and Mg^2+^, on these shells.

## 2. Results

### 2.1. The Form of the Studied Plasmid DNA

**Figure 1:**
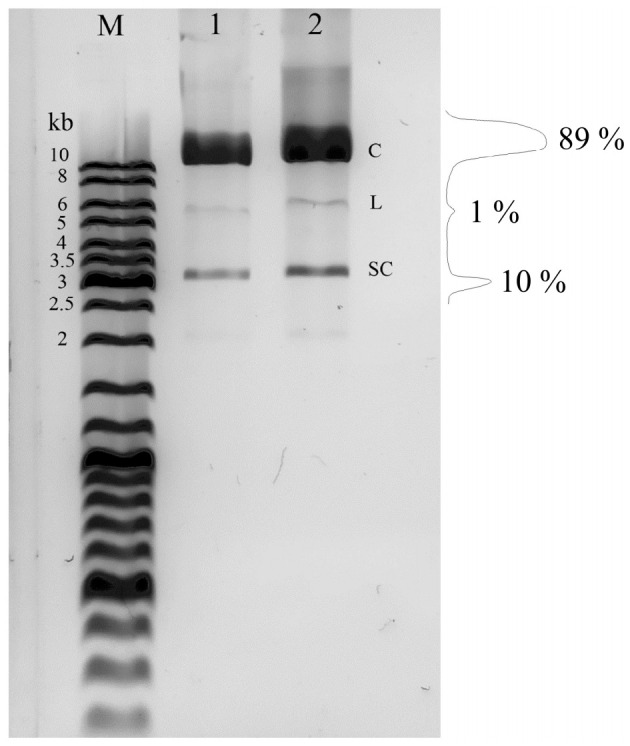
Electrophoresis of pET-11c plasmid DNA and its distribution by fractions: C—circular DNA, L—linear DNA, SC—supercoiled DNA. The original data are in Supplementary file S1.

Figure 1 shows the results of electrophoresis of the obtained plasmid DNA in 1% agarose gel. It can be seen that the studied samples contain around 90% DNA in the circular (relaxed) form.

### 2.2. Dielectric Permittivities of the Water Phase of the Studied Solutions

**Figure 2:**
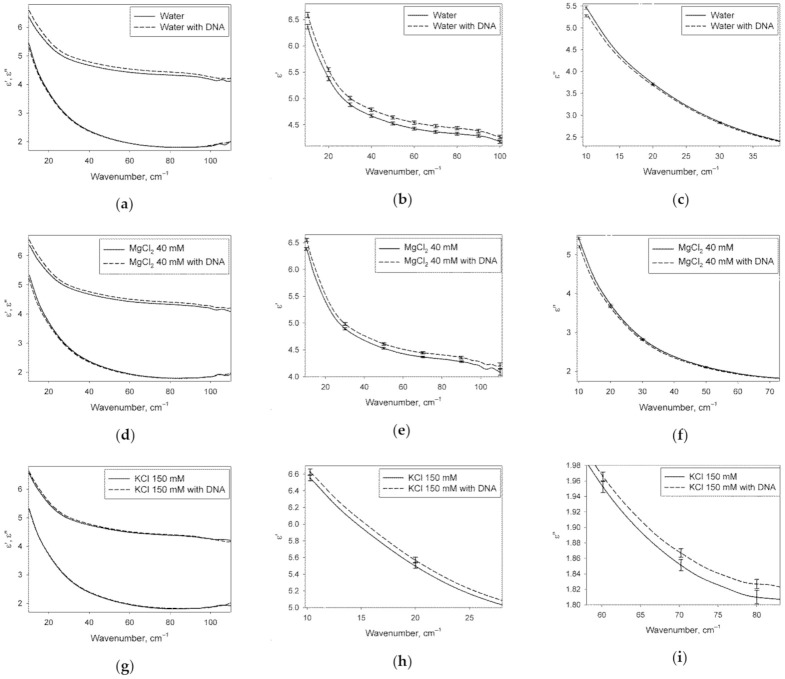
Pairwise comparison of DPs (real ε′ and imaginary ε″ parts) of the water phase of DNA solutions and aqueous DNA-free solutions with identical content of ions. The left part of the figure (**a**,**d**,**g**) shows DPs in the whole analyzed range of wavenumbers. The central and right parts of the figure (**b**,**c**,**e**,**f**,**h**,**i**) show ε′ and ε″ in the frequency regions where reliable differences were recorded. The error bars represent 95% confidence intervals. The original data are in Supplementary file S2.

Figure 2 shows DPs of the water phase of DNA solutions, obtained from experimental DPs of the DNA solutions after transformation (4), compared to DPs of the solutions of identical ionic composition without DNA.

It can be seen from Figure 2 that ε′ is elevated across almost the entire analyzed spectral range, while ε″ is decreased in the low-frequency part under the action of DNA in water and MgCl_2_ solution. In the KCl solution, ε′ is also elevated under the action of DNA, but only in the low-frequency region, and ε″ is increased in the middle of the range studied. Consequently, from the point of view of dielectric properties, water phases of DNA solutions in pure water and in 40 mM MgCl_2_ are similar to each other, but they differ significantly from the water phase of DNA solution in 150 mM KCl.

### 2.3. Parameters of the Model Dielectric Permittivity and the Percentage of Free Water Molecules in the Studied Solutions

Based on the fitting of DPs, parameters of model (5) and percentage of free water molecules (Formula (7)) were calculated. They are listed in Table 1.

The presence of DNA provides attenuation of Debye relaxation (decrease of Δ*ε*_1_) and enhancement of two other polarization processes, relaxation of free water molecules (increase of Δ*ε*_2_) and intermolecular oscillations of hydrogen-bonded water molecules (elevation of A/*ω*^2^). An increase of relaxation time *τ*_2_ and the percentage of free water molecules *n* is also observed in the presence of DNA. Elevation of *ω* and *γ* mean values is also observed, but it does not result in a statistically significant difference, hence it is not discussed further.

It is also clearly seen that the changes of the specified parameters under the action of DNA are comparable for DNA solutions prepared with water or 40 mM MgCl_2_, but are noticeably weaker for KCl solution.

### 2.4. Dielectric Permittivity of Dry DNA

**Figure 3:**
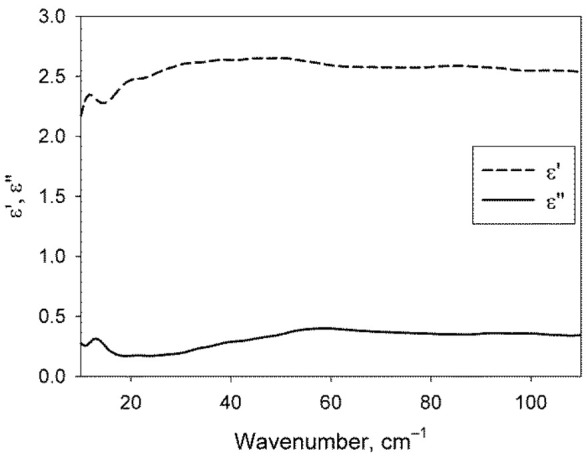
Real *ε*′ and imaginary *ε*″ parts of the DP of DNA contained in the studied solutions.

Figure 3 shows the DP of dry DNA films, which were used to calculate the DPs of the water phase of DNA solutions using Formula (4).

### 2.5. Conductivities of the Studied Solutions

Table 2 shows the DC-conductivity values of the solutions under study. These values were used to calculate the parameters of Table 1 based on the model DP (5).

## 3. Discussion

The circular form of plasmid DNA was used in this work (Figure 1). This form of DNA is present in prokaryotic (genomic DNA and plasmids) and eukaryotic (mitochondrial, plastid and non-chromosomal circular DNA) cells, as well as in the majority of DNA-containing viruses. It is interesting that even the linear form of some DNA viruses is closed into a circular molecule upon virus entry into the cell [31]. Circular DNA plays an important role in gene replication and transcription. Circular DNA is the most evolutionarily ancient, and it is the most convenient in terms of replication. Linear forms of chromosomal DNA of higher organisms appeared significantly later and required the development of auxiliary mechanisms of replication (telomeric DNA) [32]. Currently, non-chromosomal circular DNA of eukaryotes has drawn significant attention from researchers because of its important role in the regulation of expression of various genes, immune response processes, intercellular interaction and carcinogenesis [33].

DPs shown in Figure 2 characterize the water structure of the studied solutions. In the case of DNA solutions, these DPs are related to the water phase only, because the contribution of DNA itself was excluded from DPs using the effective medium model (4). A fraction of water molecules of DNA solutions belonging to hydration shells differ from undisturbed water in the structure. This is the reason for the observed differences in the DPs of DNA solutions and DNA-free solutions of the same ionic composition. It can also be seen that from the point of view of dielectric properties, the hydration shells of DNA in pure water and MgCl_2_ solutions are similar to each other, but they differ from the hydration shells in KCl solution.

The mentioned differences of DPs can be analyzed more thoroughly upon consideration of parameters calculated from DPs listed in Table 1. Let us consider the trends in each of the parameters when comparing a DNA solution and a solution without DNA. A decrease of Δ*ε*_1_ parameter of DNA solutions demonstrates depolarization processes [34,35], the most important of which in the studied solutions is the binding of water molecules into the hydration shells of DNA. The ability of DNA to bind water molecules was established by different methods [5,6,7] and the binding sites were mapped, with phosphate groups being the strongest of them. Besides decreasing Δ*ε*_1_, DNA facilitates elevation of Δ*ε*_2_ parameter (Table 1). This shows an increased amount of free water molecules [36,37,38], which is also confirmed by the direct calculation of *n* (Table 1). From the first sight, this conclusion contradicts the previous one, because we suggest both an increase and decrease of the degree of water binding at the same time. However, the simultaneous manifestation of the two mentioned tendencies was observed in other works on studying hydration shells. It was revealed by THz-TDS for a DNA solution [23], for protein solution in a partially aggregated form [27], as well as for sugar solutions [39]. In another work [40], high resolution microwave dielectric spectroscopy allowed revealing the co-existence of bound and hypermobile water molecules in the hydration shells of ATP and actin filaments. The explanation for this phenomenon, in our opinion, is the alteration of the water structure by energetically strong binding of water molecules in the first hydration layer, which does not allow the formation of undisturbed, normally bound water beyond the first hydration layer. At the same time, there is no strong binding of water molecules outside the first hydration layer. Thus, one can speak of a two-layer structure of the hydration shell, where the strong-bound (primary) shell forms the first layer and the destructured (secondary) shell forms the second layer, with undisturbed water outside of them.

Broadly speaking, the THz spectroscopy technique essentially changed the conventional idea of a hydration shell. It was found to be much thicker in biological macromolecules, consisting of not just one, but dozens of water molecule layers [29,41,42,43]. Some authors even used the term “dynamic hydration layer” [41] or “global hydration” [44], to which they did attribute not only adjacent strongly bound molecules, but also those more distant from the macromolecule, which differ from undisturbed water in their molecular dynamics measurable in the THz area. Such altered dynamics are apparently related to the emergence of a higher number of free molecules, which is reflected in the elevation of the Δ*ε*_2_ parameter in the current work.

As for the increase of A/*ω*^2^ under the action of DNA (Table 1), we can suggest the following. The A/*ω*^2^ parameter determines the contribution of intermolecular oscillations of hydrogen-bonded molecules into DP (by analogy with Δ*ε*_1_, Δ*ε*_2_ for relaxation processes). This value depends on both the number of water molecules and the number of hydrogen bonds per unit volume of the solution. Since the concentration of water molecules in the compared solutions is almost identical, a conclusion on the presence of a higher number of hydrogen bonds in the DNA hydration shells compared to undisturbed water can be drawn. An analogous result was obtained earlier in the work [30] on hydration shells of ATP molecules. We should note that the average number of hydrogen bonds with a water molecule in pure water at 25 °C comprises 3.6 of a possible 4 [45]. Hence, the expansion in the hydrogen bond number in DNA hydration shells does not exceed 10% compared to undisturbed water, which indicates small but significant differences.

The effect of DNA on water molecules is not restricted to the change of distribution of water molecules with different bonding degrees. An increased relaxation time *τ*_2_ is also observed (Table 1). The increase of this parameter has been registered earlier for DNA solutions [23], for solutions of protein in a partially aggregated form [28], as well as for suspension of DPPC liposomes in the rippled gel phase [29]. However, as well as in the mentioned papers, this experimental fact cannot be explained when using only the THz spectroscopy data. Let us put forward a hypothesis similar to the one stated in the study [28]. The structure of hydration shells contains water molecules with different hydrogen bonding degree, including some free water molecules. The existence of a free molecule is possible while it is located in some kind of a cavity composed of water molecules with fully occupied hydrogen bond vacancies. The smaller the cavity, the stronger the slowing effect it has on the orientational relaxation of free water molecules, leading to *τ*_2_ increase. It can be supposed that the average structure of these cavities in the hydration shells of DNA and some other macromolecules in certain conformations is more compact than in undisturbed water. However, such a hypothesis should be verified by methods of molecular modeling.

It is interesting to analyze the discussed regularities for DNA solutions compared to the ATP solution. An investigation devoted to studying the hydration shells of ATP by THz-TDS [30] demonstrated that the effect of ATP and Mg·ATP complex on water can be reduced to the decrease of Δ*ε*_1_ and increase of Δ*ε*_2_ and A/*ω*^2^. Similar changes of the same parameters are also observed in the current work for DNA. Let us compare quantitative changes of three parameters occurring under the action of DNA (Table 1) and ATP [30] in a 40 mM MgCl_2_ solution. For DNA, Δ*ε*_1_ is decreased 3.1 stronger, while Δ*ε*_2_ is increased 3.6 times stronger, A/*ω*^2^ is increased 1.4 times stronger, compared to ATP, when normalized by molar concentration of nucleotides. Thus, DNA and ATP have a similar effect on water structure, but the effect of DNA on all the parameters is several times stronger. It could apparently be explained by a cooperative effect. If a single nucleotide is capable of inducing certain changes in the structure of adjacent water, nucleotides arranged into a double polymer strand lead to similar changes, but they are more stable, spreading through greater volume.

Taking into account the interpretations described above, the changes of Δ*ε*_1_, Δ*ε*_2_, A/*ω*^2^ parameters under the action of DNA can be explained by the formation of three different water fractions in the hydration shell of DNA molecule, namely strongly bound water molecules, free water molecules and water molecules with increased hydrogen bonding degree. These types of water molecules exist in any water solution even in the absence of DNA, but they are present in higher quantities in the hydration layer of DNA.

The question on the distribution of the three mentioned fractions of water molecules in relation to the DNA molecule deserves special attention. It is obvious that strongly bound water molecules are arranged along the sugar phosphate backbone of DNA, because phosphate groups are the most strongly hydrated [5,6,7]. As was already mentioned, the strong binding of the first hydration layer does not allow for the arrangement of the undisturbed water layer right after it is possible. Because of that, a less structured second hydration layer can be formed. Hence, the recorded higher number of free water molecules in DNA solutions should also be distributed along the sugar phosphate backbone outside the first hydration layer.

**Figure 4:**
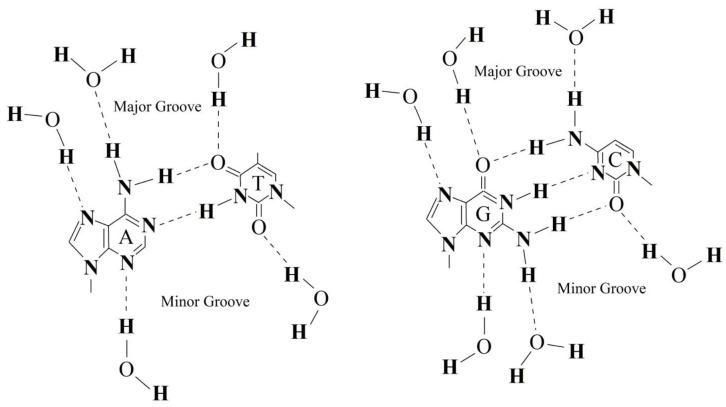
Schematic representation of water molecule hydrogen bonding in the minor and major grooves of DNA double helix. Complementary pairs of nitrogen bases, A-T (**left**) and G-C (**right**) are shown.

The fraction of water molecules with an increased number of hydrogen bonds is apparently located inside the grooves of the DNA molecule. As it is known, hydrogen bonding of water to nitrous bases occurs via endocyclic nitrogen atoms, exocyclic keto and amino groups (Figure 4). It is also known that the formation of a hydrogen bond with a water molecule leads to its increased ability to form another hydrogen bond [46]. The formation of numerous hydrogen bonds with nitrogen bases can initiate the formation of a specific layer of water molecules with an increased number of hydrogen bonds along the grooves.

Studies of DNA hydration shells by dielectric spectroscopy in the GHz region [14,18] proved the presence of a specific layer of water in the grooves, called “stringlike clusters” by the authors. It was suggested that this layer is formed via a cooperative process because it is degraded during DNA denaturation. As it was shown above, when comparing DNA and ATP, the observed formation of the DNA hydration shell structure is also cooperative, including the formation of the fraction with increased hydrogen bonding. Apparently, this fraction recorded by THz spectroscopy corresponds to the mentioned stringlike clusters registered in the GHz region.

A question on the role of ions in the formation of DNA hydration shells deserves particular attention. Various cations are known to affect the DNA structure significantly [47]. It is logical to expect their effect on the hydration shell structure. As shown above, the effect of DNA on water through the formation of hydration shells can be described by the changes of parameters Δ*ε*_1_, Δ*ε*_2_, A/*ω*^2^, *n*, *τ*_2_ during the transition from DNA-free electrolyte to the DNA solution (Table 1). All these parameters are changed in phase for the three types of solvents explored in the work, i.e., water, 40 mM MgCl_2_ and 150 mM KCl. It is notable that the changes of each parameter in water and MgCl_2_ solution are close in quantitative terms, whereas the changes of all the five parameters in KCl solution are significantly smaller. This proves that hydration of DNA is structurally close but far less prominent in KCl compared to the two other solvents. When comparing the ionic strength of all these solvents, we concluded that ionic strength is not the determining factor of the effect of salts on the hydration shells. To explain the observed effects, specific action of Mg^2+^ and K^+^ cations should be considered.

Let us consider the differences of DNA molecules in the presence and absence of Mg^2+^. It is well known that Mg^2+^ ions bind to phosphate groups via strong Coulomb interactions [47,48]. Hence, at moderate concentrations, Mg^2+^ is concentrated close to phosphate groups. In the absence of Mg^2+^, each phosphate group has a charge of −1. In this case, ion–dipole interaction and partially hydrogen bonding of the negatively charged phosphate group with positive sites of a water molecule occurs. Upon binding of Mg^2+^, the charge of the phosphate group switches from −1 to +1, and ion–dipole interaction of positive magnesium ion with negative sites of water molecule occurs. Thus, the binding of phosphates to Mg^2+^ leads to a change of water molecule orientation in the first hydration layer. Regarding affecting the water structure along the sugar phosphate backbone, there is no essential difference between these two cases. So, the conclusions on the first hydration layer strongly bound to phosphates and destroyed water structure outside this layer that we drew above do not change essentially in the case of appearance of Mg^2+^ in solution. Being localized close to phosphate groups, Mg^2+^ ions cannot strongly affect the structural parameters of water in the grooves due to the large distance. Thus, the fraction of water molecules with increased hydrogen bonding in the grooves does not undergo significant changes in the presence of Mg^2+^. As a result, we conclude that hydration shells of DNA should not strongly differ in water and MgCl_2_, which corresponds to our experimental data (Table 1).

K^+^ cation possesses twice a lower charge than Mg^2+^ and a 1.5-fold greater ionic radius [49]. As a consequence, the energy of Coulomb interaction of the phosphate group with K^+^ is three times lower than that with Mg^2+^. The K^+^ ion has also much higher translational mobility than Mg^2+^ [50]. Without any apparent localization near the DNA molecule, K^+^ ions can effectively penetrate the hydration shell of DNA near the sugar phosphate backbone as well as near the grooves. Since K^+^ is a chaotropic ion [51], it has a slight destructuring effect on water. Hence, K^+^ ions should facilitate the decrease of all three described fractions of water in the DNA hydration shells and reduce the difference between undisturbed water and the hydration shells. It can help to provide a qualitative explanation of the reason for the similar but weaker effect of DNA on the water structure in KCl compared to pure water or MgCl_2_.

The described effects of action of DNA on water structure and the specific dependence of these effects on cations can be interesting not only from the point of view of bioorganic and physical chemistry, but also from a biological point of view. The reason is that K^+^ is the main intracellular ion with a physiological concentration of about 150 mM. The work [52] showed that K^+^ ions in concentrations close to intracellular values are necessary for the formation of a correct structure of telomeric G-quadruplex DNA. Other works [53,54] demonstrated that DNA compactization is dependent on ion types, being different even for such similar cations as K^+^ and Na^+^. It might be possible that the described action of K^+^ in intracellular concentration on the DNA hydration shell is a somewhat optimizing factor important for molecular biology processes. This topic can be a subject of a separate study with a stronger accent on the biological side of the topic.

## 4. Materials and Methods

### 4.1. Preparation of Samples

Solutions of pET-11c plasmid DNA (5.7 kbp in length) at a concentration of 25 mg/mL were used. DNA solutions were prepared in three aqueous solvents: water, 40 mM MgCl_2_ (Helicon, Moscow, Russia), 150 mM KCl (Helicon, Russia). Deionized water MilliQ (Millipore, Darmstadt, Germany) was used for solution preparation.

Isolation of plasmid DNA was performed by the alkaline lysis method [55]. The integrity of the plasmid and absence of DNA degradation was confirmed by electrophoresis in 1% agarose gel in presence of 1 µg/mL ethidium bromide [56] (Figure 1). Densitometric analysis of electrophoregram was performed in ImageJ v.1.50 (National Institutes of Health, Bethesda, MD, USA). DNA concentration was estimated spectrophotometrically using a NanoDrop ND-1000 spectrophotometer (“NanoDrop Technologies”, Wilmington, DE, USA) by optical absorbance at 260 nm, taking the extinction coefficient value for 0.02 (μg/mL)^−1^ cm^−1^ [55]. DNA purity was assessed by ratios between the optical density at 260 nm (DNA), 280 nm (proteins) and 230 nm (phenolates and thiocyanates). The ratios were: A_260_/A_280_ = 2.01, and A_260_/A_230_ = 2.65, giving evidence of high purity of plasmid DNA preparations [57]. To achieve high precision of DNA concentration measurement, the solutions were diluted so that the measured optical density did not exceed 1.5.

Before the spectral measurements, the DNA solutions were vacuumed under 150 Torr for 20 minutes. This procedure was performed to get rid of excess dissolved gases and to prevent bubble formation on the windows of spectral cuvettes (see Section 4.2).

### 4.2. THz-TDS

Measurement of spectra in the terahertz region was carried out on a TPS Spectra 3000 spectrometer (Teraview, Cambridge, UK). The principle of the THz-TDS method is measurement of a time profile of electric strength E(t) of the field of a picosecond pulse passing through a sample and an analogous background signal. Based on the complex Fourier transform of E(t), absorption spectra and refraction spectra of the sample can be calculated. DP is calculated unequivocally from these two spectra without applying Kramers–Kronig relations. The details of the THz-TDS method are well studied, and they are described in, for example, [19]. The spectra were recorded over a 10–110 cm^−1^ range with 4 cm^−1^ spectral resolution. For each final spectrum, a series of 1800 measured E(t) functions were averaged. A 3-term Blackmann–Harris apodization function was used during the Fourier transform. The windows of spectral cuvettes were made of z-cut quartz. After placing a cuvette into the cuvette section of the instrument, a pause was held for 10 minutes in order to achieve stabilization of temperature and to purge the optical part of the spectrometer with dry air by FT-IR Purge Gas Generator 74-5041 (Parker Hannifin Corporation, Haverhill, MA, USA). Drying is required to exclude the influence of water vapor on the measured spectra. The sample temperature was stabilized at 25 ± 0.1 °C for all the measured samples by the thermostabilizing jacket of the cuvette.

A common approach for spectral analysis of liquid samples lies in recording the spectra of a sample in a cuvette and a blank cuvette serving as a background control. However, such an approach leads to the appearance of optical artifacts in the spectra [36]. To obtain spectra of higher quality, we applied another approach. The spectra were measured in two almost identical cuvettes differing only in the distance between the windows. The spectrum in the thicker cuvette was considered as “sample spectrum”, and the spectrum of the same solution in a thinner cuvette was considered as “background spectrum”. Two spectra differed only in the cuvette thickness; as a result, the spectrum of a solution with a thickness equal to the difference between the thicknesses of the cuvettes was obtained. More details of the measurement are described in [58].

To maintain a constant thickness of samples in the cuvettes, Teflon spacers with 50 and 100 µm thicknesses were inserted between the windows. Actually, the thicknesses can differ from nominal values. Since the measured spectra were subjected to further numerical analysis, it was important to know the thickness of a measured sample with high accuracy. Meeting that requirement, we determined accurate distances between the windows after cuvette assembly. For this purpose, an interferometric approach with an FTIR spectrometer Vertex 80v (Bruker, Ettlingen, Germany) was used. The approach is basically as follows. The spectra of blank cuvettes in the area of transparency of z-cut quartz in the near IR range, 4000–8000 cm^−1^, were recorded. The spectra contain periodic interference fringes caused by the etalon effect [59]. The distance between two neighboring interference fringes Δ*ν* on the wavenumber scale is related to the distance between windows via the following relation:

(1) $$l\left(\mu m\right)=\frac{5000}{\Delta \nu \left({\mathrm{cm}}^{-1}\right)}$$

The exact distances between the windows of cuvettes used in the present work comprised 50.30 and 100.44 µm, so that we measured the spectra of solutions with a differential thickness equal to 50.14 µm.

We should especially note that during the measurements of solutions in these cuvettes the aforementioned etalon effect can be absolutely neglected. This aspect was described in detail in our work [29], where an analogous technique of spectrum recording was used.

DPs of solutions were calculated from each pair of transmittance spectrum Tr(ν) and refraction spectrum n(ν) by the following formula [60]:

(2) $$\varepsilon \prime \left(\nu \right)=n^{2}\left(\nu \right)-{\left[\frac{\mathrm{lnTr}\left(\nu \right)}{4\pi \nu l}\right]}^{2},\;\varepsilon \prime \prime \left(\nu \right)=\frac{-n\left(\nu \right)\mathrm{lnTr}\left(\nu \right)}{2\pi \nu l},$$

where ε′ and ε″ are the real and imaginary parts of DP, ν is wavenumber, l is sample thickness (50.14 µm in the current work).

Each sample was measured at least 35 times for further averaging and statistical analysis.

### 4.3. Calculation of DP of DNA Solution Water Phase

The goal of the current work was to study the hydration shells of DNA. For this purpose, the characteristics of water in DNA solutions must be analyzed. From the point of view of dielectric properties, DNA solution is biphasic, consisting of the water phase and phase of inclusions (DNA molecules). Hence, the contribution of DNA should be subtracted from the measured DPs. To solve such problems, effective medium models establishing the relations between the DP of the biphasic system and DPs of both phases are used. Meanwhile, there is no adequate effective medium model for the common case, because a correct description of relations between DPs is impossible without consideration of certain parameters, such as the shape of inclusions. We elaborated a theoretical model of an effective medium [61] for biphasic media with fiber-like inclusions, which is applicable to DNA solutions. At low DNA concentrations, the following relation can be written [61]:

(3) $$\varepsilon_{exp}=\varepsilon_{w}+f\frac{\left(\varepsilon_{i}-\varepsilon_{w}\right)\left(5\varepsilon_{w}+\varepsilon_{i}\right)}{3\left(\varepsilon_{w}+\varepsilon_{i}\right)},$$

where $\varepsilon_{exp}$ is experimentally measured DP of DNA solution, $\varepsilon_{i}$, $\varepsilon_{w}$ are DPs of DNA and water phase, respectively, and all the three functions are complex. *f* is the volume fraction of DNA in solution, which can be calculated by multiplying the mass fraction of DNA (2.5%) by a specific volume of DNA (0.5 cm^3^/g) [62]: *f* = 0.0125. The solution of complex Equation (3), i.e., finding both real $\varepsilon_{w}^{\prime }$ and imaginary $\varepsilon_{w}^{\prime \prime }$ parts of $\varepsilon_{w}$, looks as follows:

(4) $$\varepsilon_{w}^{\prime }=\frac{1}{e}\left(a-\sqrt{\frac{3}{2}\left(\sqrt{c^{2}+d^{2}}+c\right)}\right),\;\varepsilon_{w}^{\prime \prime }=\frac{1}{e}\left(b-\sqrt{\frac{3}{2}\left(\sqrt{c^{2}+d^{2}}-c\right)}\right),\;a=\left(3+4f\right)\varepsilon_{i}^{\prime }-3\varepsilon_{exp}^{\prime }\;b=\left(3+4f\right)\varepsilon_{i}^{\prime \prime }-3\varepsilon_{exp}^{\prime \prime }\;c=3\left(\varepsilon_{exp}^{\prime 2}-\varepsilon_{exp}^{\prime \prime 2}\right)+\left(6-28f\right)\varepsilon_{i}^{\prime }\varepsilon_{exp}^{\prime }-2\left(3-14f\right)\varepsilon_{i}^{\prime \prime }\varepsilon_{exp}^{\prime \prime }+\left(3+4f+12f^{2}\right)\left(\varepsilon_{i}^{\prime 2}-\varepsilon_{i}^{\prime \prime 2}\right)\;d=\left(6-28f\right)\varepsilon_{i}^{\prime \prime }\varepsilon_{exp}^{\prime }+6\varepsilon_{exp}^{\prime }\varepsilon_{exp}^{\prime \prime }+\varepsilon_{i}^{\prime }\left(\left(6-28f\right)\varepsilon_{exp}^{\prime \prime }+\left(6+8f+24f^{2}\right)\varepsilon_{i}^{\prime \prime }\right)\;e=2\left(5f-3\right)$$

Functions $\varepsilon_{i}^{\prime }$ and $\varepsilon_{i}^{\prime \prime }$ included in Formula (4) were determined by measuring the spectra of dry DNA films, as it was described in Subsection “Preparation of dry DNA films and measurement of their DPs”.

### 4.4. Analysis of Water Solution DPs

The following model DP was used to describe water solutions:

(5) $$\varepsilon_{mod}=\frac{\Delta \varepsilon_{1}}{1-i\omega \tau_{1}}+\frac{\Delta \varepsilon_{2}}{1-i\omega \tau_{2}}+\frac{A}{\omega_{0}^{2}-\omega^{2}-i\omega \gamma }+\varepsilon_{\infty }+i\frac{\sigma_{0}}{\varepsilon_{0}\omega },$$

where $\tau_{1}$ and $\Delta \varepsilon_{1}$ are time and strength of Debye’s relaxation of water molecules [63,64], $\tau_{2}$ and $\Delta \varepsilon_{2}$ are time and strength of relaxation of free or weakly bound water molecules [36,37,38], A, $\omega_{0}$, γ are amplitude, resonance frequency and damping parameter of intermolecular stretching oscillation of water molecules bound by hydrogen bonds [65,66], $\varepsilon_{\infty }$ is high-frequency DP (parameter describing the contribution of all higher-frequency polarization processes in water), $\sigma_{0}$ is dc-conductivity of solution, $\varepsilon_{0}$ is dielectric constant, ω is cyclic frequency, *i* is an imaginary unit.

Model DP (5) takes into account all the basic types of molecular dynamics of water in the terahertz region. It is described by 9 parameters that could be calculated by fitting the model DP to experimental DP. To reduce the uncertainty of the fitting procedure, we reduced the number of varied parameters of model (5) as described below.

Parameter $\sigma_{0}$ was measured separately (see the section “Measurement of solution conductivity”). Parameter $\varepsilon_{\infty }$ was equal to 2.5, this is the parameters specific for water solutions at frequencies around $\omega_{0}$. Maximum of Debye relaxation band (the first term in (5)) is around 0.6 cm^−1^ [67]. It is far from the analyzed frequency range of 10–110 cm^−1^, where only the high-frequency edge of the mentioned band appears. Debye relaxation is very sensitive to the hydration process. $\Delta \varepsilon_{1}$ decreases, whereas $\tau_{1}$ increases upon binding of water molecules in solution. Both events are reflected in the terahertz region as attenuated absorption at the low-frequency edge of the region. In this relation, there is no necessity to take both parameters into account independently. We set $\tau_{1}$ value at 8.28 ps, which is specific for pure water at 25 °C [67]. As a result, only six independent values of the initial nine parameters of the model (5) were left: $\Delta \varepsilon_{1}$, $\Delta \varepsilon_{2}$, $\tau_{2}$, A, $\omega_{0}$, γ, which were calculated via fitting.

The fitting criterion was minimization of *s*:

(6) $$s=\frac{1}{N}\sqrt{\sum_{i=1}^{N}\left[{\left(\frac{\varepsilon_{mod}^{\prime }\left(\nu_{i}\right)-\varepsilon_{exp}^{\prime }\left(\nu_{i}\right)}{\varepsilon_{mod}^{\prime }\left(\nu_{i}\right)}\right)}^{2}+{\left(\frac{\varepsilon_{mod}^{\prime \prime }\left(\nu_{i}\right)-\varepsilon_{exp}^{\prime \prime }\left(\nu_{i}\right)}{\varepsilon_{mod}^{\prime \prime }\left(\nu_{i}\right)}\right)}^{2}\right]},$$

“*mod*” and “*exp*” designate model and experimental DPs, *N* = 250 is the number of points in the spectrum. Fitting was performed for each pair of functions, $\varepsilon_{exp}^{\prime }\left(\nu \right)$ and $\varepsilon_{exp}^{\prime \prime }\left(\nu \right)$. The mean value of *s* comprised 0.001, and it did not exceed 0.0019 among all the separate calculations, which confirms the high accuracy of fitting.

Using the calculated model parameters (5) and absolute temperature of the samples (T = 298.15 °K), the percentage of free water molecules was estimated by formula [29,68]:

(7) $$n=\frac{2.6\times 10^{-3}\Delta \varepsilon_{2}T}{\left(\Delta \varepsilon_{2}+\varepsilon_{\infty }+\frac{A_{1}}{\omega_{1}^{2}}+2\right)\left(\varepsilon_{\infty }+\frac{A_{1}}{\omega_{1}^{2}}+2\right)}.$$

### 4.5. Preparation of Dry DNA Films and Measurement of Their DPs

The requirement to know DP of DNA is emphasized in the section “Calculation of DP of DNA solution water phase”. DNA spectra were measured in films obtained by drying DNA solutions on z-cut quartz windows under 1 mbar vacuum. Visible drying occurred in several minutes, but the samples were kept under vacuum for 14 h to achieve maximal possible dehydration degree.

Transmission and refraction spectra of the films were measured on TPS Spectra 3000 spectrometer in a dried cuvette chamber. As a background spectrum, we used the spectrum of a clean z-cut quartz window. To calculate DP of dry DNA by Formula (2), film thickness l should be known. It was measured separately on an LSM 510 META (Carl Zeiss, Germany) confocal microscope in reflection mode with 488 nm Ar laser, dry objective 10×/0.30 Plan-Neofluar, aperture size 0.1 of the Airy disk. Scanning across the film thickness allowed recording both its surfaces by the burst of the intensity of the registered laser illumination. On this basis, film thicknesses were measured.

### 4.6. Measurement of Solutions Conductivity

Measurement of conductivity $\sigma_{0}$ included in Formula (5) was performed with high accuracy on Zetasizer nano ZS (Malvern, Malvern, UK) in zeta potential measurement mode. The measurements were taken in capillary cuvettes with DTS1060 electrodes (Malvern, UK) at 25 °C.

## 5. Conclusions

The analysis of complex dielectric permittivity of DNA solutions obtained by THz-TDS enabled us to describe certain peculiarities of the DNA hydration shells. The obtained data show that the hydration shells differ from undisturbed water by the presence of strongly bound water molecules, a higher number of free molecules and an increased hydrogen bond count. Strongly bound water molecules are formed upon interaction with phosphates. Free water molecules are arranged along the sugar phosphate backbone beyond the first layer of strongly bound molecules. Molecules with elevated hydrogen bond count are contained in the grooves of the DNA molecule. The presence of Mg^2+^ cations does not lead to strong changes in DNA hydration shells, whereas K^+^ cations partially destroy them. The obtained results significantly expand the existing knowledge about DNA hydration and demonstrate a high potential for using the THz time-domain spectroscopy method.

## Figures and Tables

**Table 1:** The parameters of model DP (5) and the percentage of free water molecules *n* (7) calculated for all the studied solutions.

Title ^1^	Δ*ε*_1_	Δ*ε*_2_	*τ*_2_, ps	*ω*, cm^−1^	A/*ω*^2^	*γ*, cm^−1^	*n*, %
Water	**68.86 ± 0.81**	**2.691 ± 0.042**	**0.316 ± 0.006**	207.2 ± 4.3	**1.702 ± 0.019**	196.5 ± 11.1	**3.78 ± 0.04**
Water with DNA	**61.70 ± 0.97**	**3.088 ± 0.095**	**0.346 ± 0.009**	217.3 ± 7.5	**1.831 ± 0.037**	215.4 ± 16.2	**4.01 ± 0.07**
MgCl_2_ 40 mM	**67.59 ± 0.63**	**2.709 ± 0.036**	**0.318 ± 0.004**	209.2 ± 4.8	**1.710 ± 0.016**	201.9 ± 11.1	**3.79 ± 0.04**
MgCl_2_ 40 mM with DNA	**60.55 ± 0.71**	**2.992 ± 0.046**	**0.337 ± 0.005**	218.1 ± 6.3	**1.809 ± 0.026**	216.9 ± 15.0	**3.95 ± 0.04**
KCl 150 mM	**63.11 ± 0.93**	**2.953 ± 0.066**	0.329 ± 0.007	211.6 ± 5.3	1.771 ± 0.025	201.2 ± 11.8	**3.95 ± 0.06**
KCl 150 mM with DNA	**59.44 ± 0.66**	**3.105 ± 0.040**	0.335 ± 0.005	217.3 ± 4.0	1.807 ± 0.025	215.5 ± 9.2	**4.05 ± 0.03**

**^1^** The data are presented as pairwise comparison of the water phase of DNA solutions and aqueous solutions without DNA with analogous ion content. The dispersion shown covers 95% confidence intervals. The parameters that are significantly different for the compared solutions are shown in bold type.

**Table 2:** dc-conductivity values of all the studied solutions at 25 °C.

Title 2						
Solution	Water	Water with DNA	MgCl_2_ 40 mM	MgCl_2_ 40 mM with DNA	KCl 150 mM	KCl 150 mM with DNA
Conductivity, S/m	0	0.502	0.825	1.466	1.740	3.325

## Data Availability

Not applicable.

## Supplementary Materials

The following are available online at https://www.mdpi.com/article/10.3390/ijms222011089/s1.
