# Supplementary figures and images for: Hydration Shells of DNA from the Point of View of Terahertz Time-Domain Spectroscopy
Source: Int J Mol Sci. 2021 Oct 14;22(20):11089. doi: 10.3390/ijms222011089 (PMC8538832; doi:10.3390/ijms222011089)

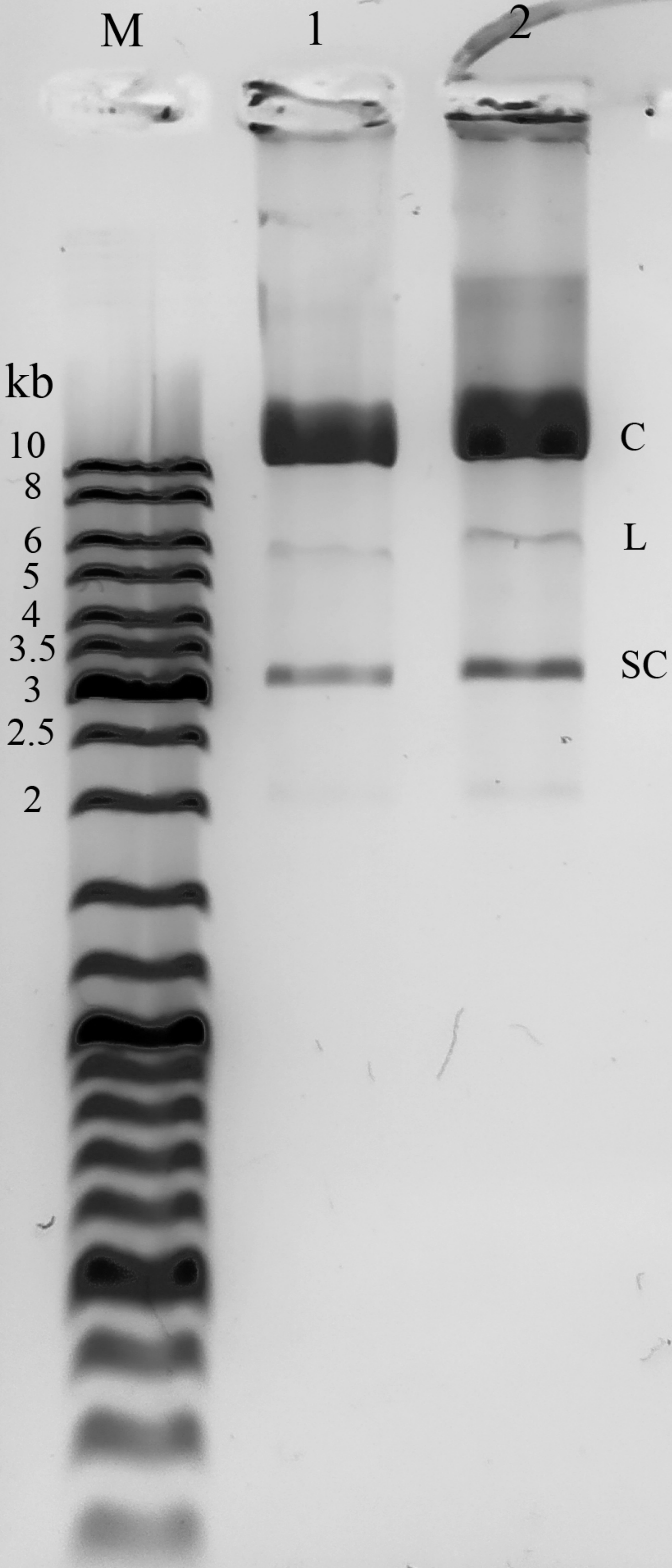

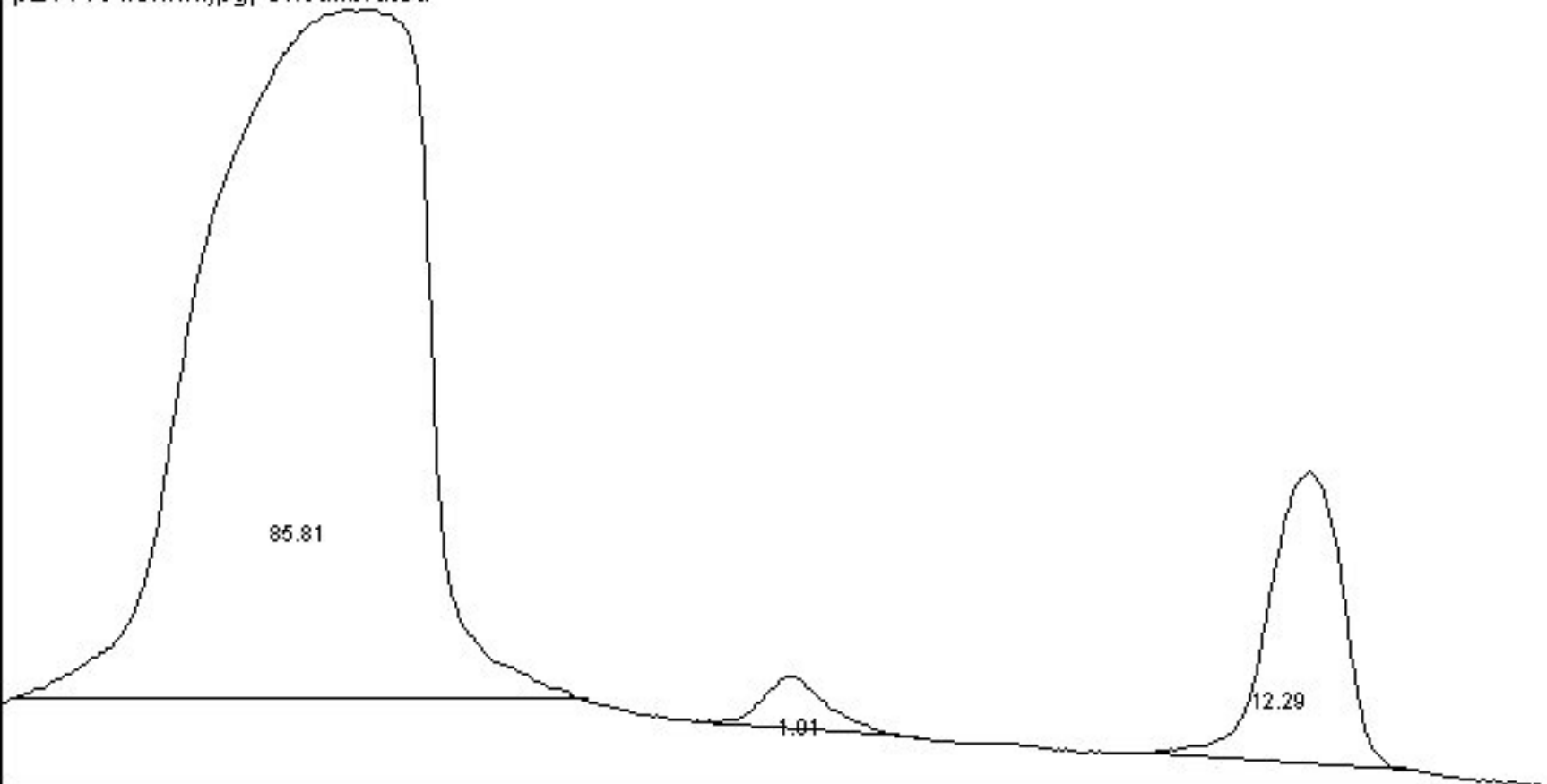

Supplement: Supplementary file 1 [file ijms-22-11089-s001.zip › Supplementary file S1.pdf]
